# Effects of a Technology-Supported Decision, Reflection, and Interaction Approach on Nursing Students’ Learning Achievement and Self-Efficacy in Professional Training: A Pilot Study

**DOI:** 10.3390/healthcare11081164

**Published:** 2023-04-18

**Authors:** Gwo-Jen Hwang, Hsiu-Ju Jen, Ching-Yi Chang

**Affiliations:** 1 Graduate Institute of Digital Learning and Education, National Taiwan University of Science and Technology, Taipei 10607, Taiwan; 2 Yuan Ze University, Taoyuan 32003, Taiwan; 3 School of Nursing, College of Nursing, Taipei Medical University, Taipei 11031, Taiwan; 4 Department of Nursing, Taipei Medical University-Shuang Ho Hospital, New Taipei City 23561, Taiwan

**Keywords:** decision making, expert system, mobile learning, nursing education, professional training

## Abstract

In professional training, it is important to provide students with opportunities to make judgments on practical cases. However, most training courses are conducted in a one-to-many teaching mode, and it is not easy to consider the needs of individual students. In this study, a technology-supported Decision, Reflection, and Interaction (DRI)-based professional training approach is proposed to cope with this problem for those courses aiming at fostering students’ competence in making correct judgments when facing real cases. To verify the effectiveness of the proposed method, an experiment was conducted. Two classes of 38 students from a nursing school were the participants. One class was an experimental group using the DRI-based professional training approach, and the other class was the control group using the conventional technology-assisted training approach. The experimental results showed that applying the proposed approach significantly improved the students’ learning achievement and self-efficacy more than the conventional technology-assisted approach. In addition, based on the interview results, the students generally believed that learning through the DRI-based professional training approach benefited them from several perspectives, including “increasing the value of activities”, “enhancing the planning and expensive capacity of conspicuous approaches”, “promoting decision-making”, “improving learning reflection”, and “providing students with personalized interaction”.

## 1. Introduction

In the 21st century, the content of professional training, such as medical and nursing education, is constantly undergoing innovation and change; therefore, improving the knowledge and decision-making abilities of students is a crucial goal in vocational education [1]. For example, caring for the health of newborns is even more challenging in the face of various infectious diseases, such as during the COVID-19 pandemic [2,3]. In particular, there are several challenges to neonatal vaccination in Taiwan. For example, parents generally resist having their newborns vaccinated [4]. Moreover, owing to the low birth rate, the nursing staff generally lacks experience dealing with neonatal vaccinations [5]. As the considerations for vaccinations for newborns are quite different from those for adults, additional training is required to foster the nursing staff’s ability to explain the neonatal vaccination process and resolve parents’ concerns.

However, in many professional training courses, students are mostly taught using traditional teaching methods. Because such methods generally provide few opportunities for students to participate in making judgments and receiving feedback in the process of dealing with practical cases, it is difficult for them to think deeply about the professional knowledge issues at each stage [6]. From a constructivist perspective, which emphasizes that knowledge can only be actively constructed from learners’ experiences and social discourses, it is very important for students to participate in an interactive learning mode to promote knowledge construction. In recent years, some scholars and instructors have adopted computer technologies in professional training programs; however, without proper supports, the training outcomes may not be as good as expected [7]. In response to this problem, researchers have pointed out that if experts’ past knowledge and experience of making judgments on practical cases can be constructed into a computerized expert system, this system could assist students in solving the problems encountered in the case judgment process through interactive guidance [8,9,10]. An expert system refers to a computer system that can imitate the reasoning procedures of human experts and use the collected expert knowledge to solve problems in a specific field [11]. Scholars have further pointed out that in the process of interacting with an expert system, students can refer to the information provided by the system and compare it with and reflect on their original knowledge, thereby constructing new knowledge and strengthening the cognitive structure [12].

Several researchers have attempted to use expert systems and natural language to improve student learning efficiency [13,14]. In various educational applications, an expert system generally serves as a tutor or advisor in a specific subject area to guide individual students to learn [15]. Compared with conventional technology-assisted teaching methods, expert systems not only allow learners to actively explore and construct knowledge through an interactive process but also provide individual feedback and guidance to improve learning effects [16]. On the other hand, though researchers have emphasized the importance of guiding students to learn with the assistance of expert systems [10,17], without a proper learning model, students’ learning performance when using expert systems in educational settings could be disappointing [18].

Consequently, the current study proposed a Decision, Reflection, and Interaction (DRI)-based mobile professional training approach that integrated an expert system with mobile technology. By referring to the literature, the study used a quasi-experimental design to answer the following research questions to evaluate the effectiveness of the proposed approach in professional training courses:

(1) What are students’ learning experiences with the DRI-based mobile professional training approach compared with conventional technology-assisted teaching?
(2) Compared with conventional technology-assisted teaching, can the DRI-based mobile professional training approach promote students’ learning achievement in a course on neonatal vaccination knowledge?
(3) Compared with conventional technology-assisted teaching, can the DRI-based mobile professional training approach improve students’ self-efficacy in a course on neonatal vaccination knowledge?

## 2. Literature Review

Artificial intelligence (AI) refers to the research field that aims to develop computer systems that can imitate human intelligence, such as inference, prediction, and decision-making [10,19]. There are several AI technologies, such as neural networks, deep learning, symbolic machine learning, natural language processing, and statistical analysis, which have been successfully applied to various applications, including speech recognition, image recognition, expert systems, intelligent games, intelligent tutoring systems, prediction, and decision-making [20]. Tang et al. [19] pointed out that the application of AI in education includes analysis, prediction, guidance, assessment, and adaptive learning. Hwang, Sung, Chang, and Huang [21] further noted that in educational applications, AI can play the roles of smart teachers, smart learning companions, and smart students. 

According to the literature, expert systems are also called knowledge-based systems, knowledge systems, or intelligent systems [22,23]. By definition, an expert system refers to the computer system that employs an inference mechanism to make decisions or suggestions for the cases to be resolved based on the domain expertise stored in the knowledge base; that is, an expert system is a system that employs professional knowledge and an inference mechanism to simulate the decision-making process of domain experts [8,24]. Scholars have indicated that an expert system has several characteristics: (1) it can provide complex and judgmental knowledge in real situations; (2) it has the ability to solve problems with high efficiency; (3) it can solve problems in a specific field; and (4) it has reasonable inference and explanation functions [25,26]. In educational usage, when a learner enters the descriptions of a case, the expert system can provide advice or recommendations for that case [27].

Many scholars have used expert systems to solve various educational problems. For example, Goksu [28] applied a web-based expert system to evaluate the cognitive level of students from the perspective of Bloom’s taxonomy. Wanyan, Chen, and Olowokere [29] reported that the personalized guidance provided by an expert system could improve students’ learning efficiency. Hwang, Sung, Chang, and Huang [21] found that an expert system that considered emotional and cognitive factors could further improve students’ learning achievement. In addition, some expert systems can even use a machine learning mechanism to interact with learners to improve the system’s inferential judgment ability; for example, López-Úbeda et al. [30] added a machine learning mechanism to an expert system to analyze students’ radiation knowledge. With the machine learning mechanism, the performance of the expert system could be improved after receiving feedback on the correctness of the decision it made.

On the other hand, scholars have indicated that few studies have attempted to apply expert systems to professional training [31]. This could be due to the fact that implementing expert systems is a high-tech and challenging mission [32]. Anwar [33] further indicated another challenge of using digital technologies in educational settings, namely, that a proper learning design is needed for guiding students to use technologies to solve problems during the learning process. Without a clear guiding model, it could be too difficult for school teachers or instructors to design expert system-supported learning activities or training programs. Therefore, this research aimed to propose a model to demonstrate how an expert system can be used in professional training.

## 3. Decision, Reflection, and Interaction (DRI)-Based Professional Training Model

**Figure 1:**
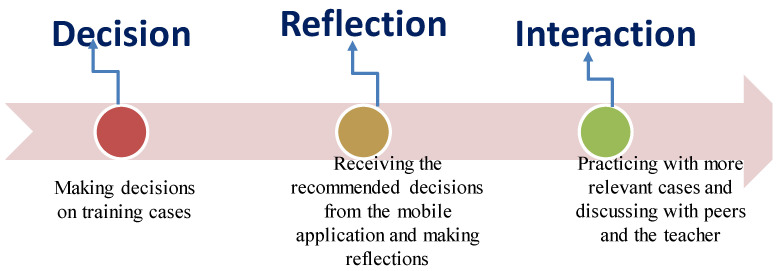
The DRI-based professional training model.

Figure 1 shows the DRI-based professional training model, which consists of three learning stages: Decision, Reflection, and Interaction. In the Decision stage, students are guided to make decisions on the cases provided by the teacher based on what they have learned. In the Reflection stage, they are asked to examine and make reflections on the decisions they have made by making inquiries using a mobile application (i.e., the expert system). Following that, in the Interaction stage, the students are guided to practice with more cases and discuss what they have learned with their peers and the teacher.

In the DRI-based professional training model, the expert system plays an important role. It needs to contain the required case data and decision-making techniques based on domain expertise [8]. In addition, it is important that the system be capable of interacting with learners using a natural language interface [34]. With the decision-making ability and the natural language interface, the system can make judgments and provide suggestions to individual learners during the professional training process. 

**Figure 2:**
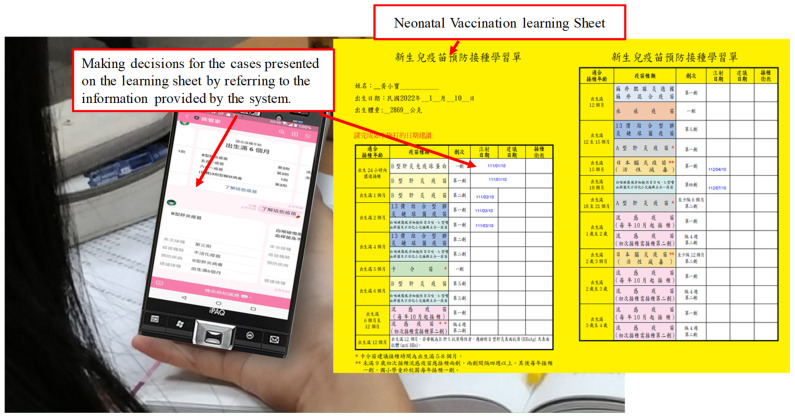
Example of the learning scenario in the Decision stage.

In the Decision stage, as shown in Figure 2, after the teacher explained the neonatal vaccine curriculum, the students were asked to make decisions on the weekly neonatal cases presented on the learning sheet. They can read the supplementary content provided by the system on their mobile phones while making decisions.

**Figure 3:**
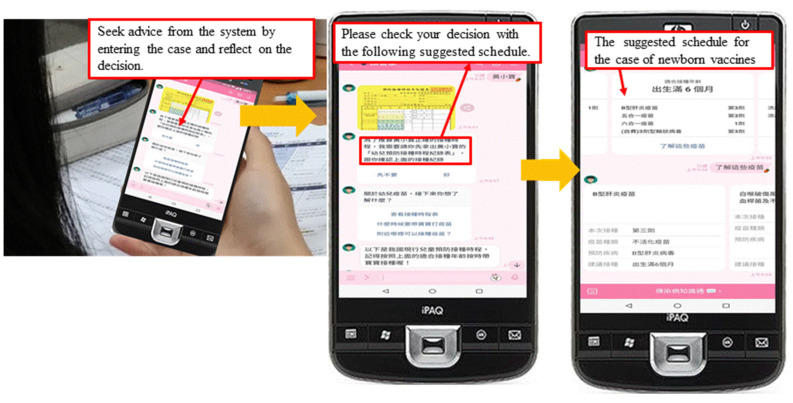
Example of the Reflection stage.

As shown in Figure 3, in the Reflection stage, students can seek advice from the system by entering the descriptions of the cases they are handling. The system provides detailed suggestions related to the case, such as post-vaccination protection and care priorities in relation to the type of vaccine given to newborns. By comparing the suggestions with their own decisions, students are guided to make reflections regarding the details they may have missed.

**Figure 4:**
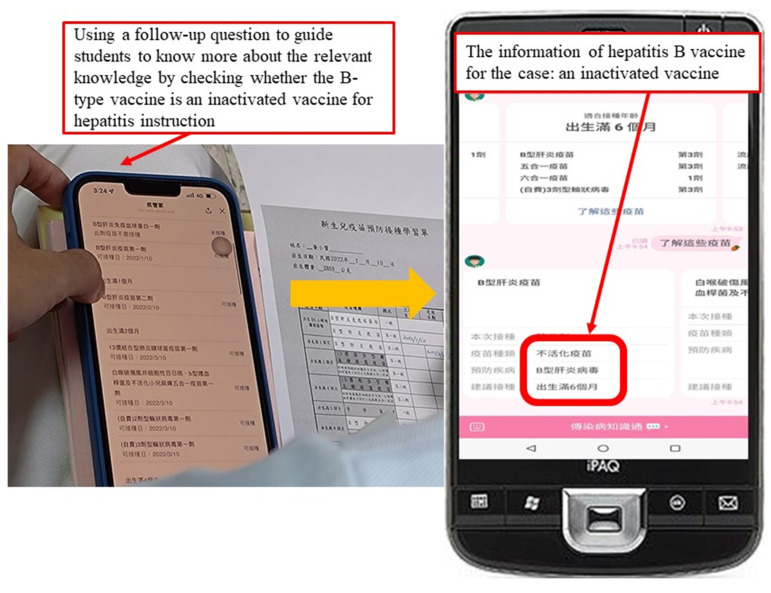
Example of the Interaction stage.

During the Interaction stage, as shown in Figure 4, students were guided to extend their learning about vaccines by answering some follow-up questions on the learning sheet, such as the timing and type of vaccines to be administered next, statutory vaccine expertise, and care priorities for disease infection control, as well as special problems to be taken into account during the COVID-19 epidemic. 

## 4. Materials and Methods

### 4.1. Method

This research used a quasi-experimental design with pretests and posttests to verify the differences in the learning performance, self-efficacy, and learning experience of students who learned through the DRI-based professional training model and those who learned through conventional technology-assisted teaching methods. The selected subject unit was “vaccine management capabilities for neonatal vaccination”, which is an important issue for protecting pregnant women and newborns from infection, in particular during the COVID-19 epidemic. Knowledge of neonatal vaccination and the schedule of vaccination has become part of the basic clinical requirements for nursing school students. The study was conducted from February to August 2021 at a nursing school in northern Taiwan. The experiment was conducted in an internship setting. The activity was not part of the student’s degree but was a component of their internship before entering the workplace. Moreover, ethical approval was obtained from the school’s research ethics committee, and each participating student completed a consent form for participation in the experiment.

### 4.2. Participants

Thirty-eight students with an average age of 21 were recruited from two classes. Training students in a course on neonatal vaccination, which is a basic compulsory course in nursing programs, is an important part of obstetrics and pediatric nursing training. Before the experiment, the students signed a consent form, which informed the participants of the objectives and reasons for the study. The students were also informed that the experiment would not affect their scores in the program, and they were allowed to withdraw from the experiment at any time. After introducing the objectives and reasons for the study, all of the students agreed to participate in the experiment. 

One class with 20 students was then selected as the experimental group, using the DRI-based mobile professional training approach to learn about neonatal vaccination. The other class, with 18 students, was the control group, which used conventional technology-assisted teaching methods. All students were taught by the same teacher, and no participants had participated in neonatal vaccination courses using the mobile professional training approach before the training program.

### 4.3. Experimental Process

To reflect the reality of teaching, the experiment was conducted following the original schedule of the selected course unit. In the first week, the teacher introduced basic knowledge on neonatal vaccinations and conducted questionnaire surveys and pretests to understand the students’ prior knowledge and opinions. In the second week, the teacher introduced the case that needed to be addressed. Following that, the experimental group used the DRI-based mobile professional training approach to learn about neonatal vaccination and complete the learning form, which contained a list of learning tasks. On the other hand, the students in the control group learned with the conventional technology-assisted teaching mode; that is, they were guided to use mobile devices to search the Internet to complete the same learning tasks. After the class activities, the two groups of students were required to take a post-learning performance test and a self-efficacy questionnaire survey. In addition, interviews were conducted with the experimental group and the control group.

### 4.4. Measurement Tools

The neonatal vaccination test was designed by two experienced obstetrics and pediatrics teachers with more than 10 years of clinical obstetrics and pediatrics teaching experience. The pretest was designed to evaluate the students’ prior knowledge of neonatal immunization, and the posttest was used to evaluate the learning effectiveness of the students’ neonatal immunization course activities. An example question is “When is the neonatal varicella vaccine recommended? (1) At birth, (2) 0–6 months, (3) 12 months, or (4) 12–18 months” (the answer is 12 months). The test included 10 multiple-choice questions, with a total possible score of 100 points. The Kuder Richardson-20 (KR20) values of the pretest and posttest were 0.80 and 0.78, respectively.

The self-efficacy measurement tool was constructed based on a questionnaire developed by Pintrich et al. [35] using a 5-point Likert scale. The Cronbach’s α value of the original questionnaire was 0.93, while the internal consistency of the self-efficacy items in this experiment was 0.90. These items assessed students’ self-efficacy, such as “I believe I will get excellent grades on this assignment” and “I believe I can understand the most complicated part of the teacher’s teaching in this course”.

In addition, interviews were conducted to analyze students’ learning experiences based on the viewpoints proposed by [36]. The content of the interview outline was as follows: What is the difference between the learning method for neonatal vaccination knowledge based on the DRI-based mobile professional training approach and the conventional technology-assisted teaching method? Do you think the DRI-based mobile professional training approach is useful? Why? Are you serious about learning about neonatal vaccination? Does this learning method help you? In general, what are the advantages and disadvantages of the learning method for neonatal vaccination knowledge based on the DRI-based mobile professional training approach? Do you want to learn again in the future using the DRI-based mobile professional training approach for neonatal vaccination knowledge? Would you recommend this learning method to your family and friends? 

## 5. Results

In this study, SPSS was used for analysis. Because the sample size was less than 100, academic performance and self-efficacy were tested by the Shapiro-Wilk normality test. From the test results, it was found that the *p*-values of the two variables were greater than 0.05, so the null hypothesis was accepted; that is, the academic performance and self-efficacy scores were in line with a normal distribution.

### 5.1. Interviews

To investigate the students’ learning responses to the DRI-based mobile professional training approach, the researchers conducted a 50-minute focus group interview. We randomly chose six participants from the experimental group and six from the control group to be interviewed. A researcher who had 20 years of experience in obstetric training conducted the interview. She was an assistant professor at a nursing department of a university at the time of the study. The interview was conducted in a meeting room. Before the coding process, the interview process was recorded, and a verbatim draft was created to ensure that the collected data were correct.

The interview results were coded using the grounded theory approach [37]. The interview results show that the participants in both groups generally had positive perceptions of the training program, and no negative feedback was found.

Table 1 shows the analysis results of the interview, including the themes, coding items, and number of occurrences. This condition only happened with the investigational group data: after the replication and evaluation, the generated educational benefits included “increasing the value of activities” [36] and “enhancing the planning and valuable capacity of conspicuous approaches” [37].

(1) Interview results of the experimental group

The interview results showed that the experimental group students generally believed that the DRI-based mobile professional training approach had three advantages, namely, “increasing the value of activities”, “enhancing the planning and valuable capacity of conspicuous approaches”, “promoting decision making”, “improving learning reflection”, and “providing students with personalized interaction”.

In terms of “increasing the value of activities”, examples of student responses are shown as follows:

“The training model based on the expert system makes me feel happy and helps me understand neonatal vaccination and vaccine-related knowledge. This system also provides exciting and easy-to-understand content, making me more willing to use this method to learn or teach family members” (SIS3-20210111).

“I agree with the learning method based on the DRI-based mobile professional training approach and support the curriculum based on the scientific theory of neonatal vaccine knowledge. This technological aid has enabled me to build confidence in future clinical professional workplace health education” (SIS3-202010111).

“In the course activities for neonatal vaccination knowledge, the adopted professional training approach makes me feel happy and involved because this is the first time I have used this interesting method” (RRW-202010110).

Regarding “enhancing the planning and valuable capacity of conspicuous approaches”, students generally said that the training model based on the expert system made them highly motivated, deepened their understanding of neonatal vaccines, and enabled them to obtain more neonatal vaccination knowledge regarding different vaccines, preventive measures, and techniques. Students in the experimental group generally believed that using the DRI-based mobile professional training approach to learn about neonatal vaccination could motivate them to understand and collect relevant information on new-born vaccination knowledge.

In terms of “promoting decision-making”, examples of student responses are shown as follows:

“The learning task for neonatal vaccination knowledge allows me to have a more in-depth understanding of the impact of different growth months and treatments for each newborn. Using the DRI-based mobile professional training approach to learn makes me more focused on the in-depth problem” (SIS1-20210110).

“To answer the family’s questions about neonatal vaccination knowledge in clinical practice, I need to accurately understand the concept of neonatal vaccinations instead of asking family members to find answers online. I didn’t know that there was a training model based on an expert system, and I had never practiced and learned in a similar technological environment. It was fresh and interesting” (SIS2-20210110).

In terms of “improving learning reflection”, examples of student responses are shown as follows:

“This is a very practical method. The professional and rich neonatal vaccination content and good interactive functions in the training model based on the expert system are very beneficial for the neonatal vaccination education of mothers and their families. It allows me to learn to improve my professional abilities and have confidence in health education, and it is different from conventional technology-assisted paper vaccination schedules and record sheets; in addition, in the future clinical environment, I will not be afraid of being asked about neonatal vaccination” (SIS1-20210110).

“This DRI-based mobile professional training approach can help me further explore neonatal vaccine knowledge. I hope that in the future clinical work environment of obstetrics and pediatrics, there will be more opportunities to adopt a DRI-based mobile professional training approach for neonatal vaccine knowledge and health education methods to guide mothers and their families to learn. This system is beneficial and interesting” (SIS2-20210110).

As for “providing students with personalized interaction,” the students generally stated that adopting the DRI-based mobile professional training approach helped them clarify misconceptions by providing instant responses or directions, and therefore stimulated their confidence in learning the neonatal vaccination knowledge well. For example, “The DRI-based mobile professional training approach provides integrated knowledge and tasks for learning about vaccinations and their administration from birth to before school. It can also help learners immediately correct misunderstandings of the vaccination schedule and improve mothers’ and families’ knowledge of and ability to take care of the newborn’s health. It is easy to enter the learning environment of the system through a mobile phone or the network platform of a vehicle” (RRW-20210111).

In the above interviews, most students thought that the DRI-based mobile professional training approach could help them learn and simulate the actual issues of health education in the clinical workplace to prepare for entry into the clinical environment. A DRI-based mobile professional training approach is suggested; its built-in functions can include image recognition to help students, medical staff, or the general public identify the type of vaccine. A comparison of the two groups showed that the students who learned about neonatal immunization through the technology-assisted expert system could use the system to review at any time to remember the learning content easily and could apply this system to explore and solve problems. The students in the control group only memorized part of the learning content and needed to work hard to solve practical problems in other ways. The conclusion is that adopting a DRI-based mobile professional training approach for neonatal vaccination interaction can help students connect what they have learned with actual clinical cases, thereby helping them think about and improve their professional knowledge and ability.

(2) Interview results of the control group

The interview data showed that most of the control group students considered conventional technology-assisted teaching to be a direct and simple way of delivering knowledge. In the meantime, they indicated that in such a lecture-based teaching approach, they usually did not think in depth, and several students stated that they needed to relearn a large portion of the program when dealing with real-world cases since they only tried to memorize what the teacher had taught, rather than thinking how to apply the information in the conventional technology-assisted teaching mode.

“After attending the neonatal vaccination course, I still could not answer some related questions with certainty when faced with the questions of the mother and her family. For example, I cannot clearly state the relationship between the birth months of newborns and the precautions for vaccination” (SIS10-20210112).

“When facing an actual neonatal vaccination, could I begin to think deeply? After learning the content, I only remember that live vaccines may affect the production of antibodies in newborns, so they cannot be administered at the same time” (SIS13-20210112).

“The teacher introduced the knowledge of neonatal vaccination in detail in the classroom, and I can also remember most of the learning content. However, I still feel no self-confidence after studying because it seems far from the actual clinical application” (SIS12-20210112).

“In the epidemic season, based on the CDC or the evidence in real time, we may not know the new neonatal immunization plan or changes to the clinical health education method” (SIS14-20210112).

According to comprehensive interview data regarding self-efficacy, the students believed that using the DRI-based mobile professional training approach could help them determine the time of vaccination by providing immediate feedback or guidance, thereby enhancing their confidence in learning about neonatal vaccines. Most of the students in the control group believed that conventional technology-assisted classroom teaching was a direct and simple way of disseminating knowledge. At the same time, they said that in this lecture-based teaching model, they usually did not want to think deeply, nor did they want to fully interact with the teacher. Some students also said that when dealing with actual clinical cases in the workplace, they would need to relearn most of the knowledge on newborn vaccinations because in school classrooms they just wanted to remember what the teacher taught to complete the school examinations and obtain results rather than thinking about how to apply that knowledge to solve problems. In addition, conventional technology-assisted teaching methods may not provide the opportunity to learn some of the latest knowledge related to specific diseases or vaccines in the classroom.

### 5.2. Learning Achievement

In this study, pretest learning achievement was regarded as a common variable, and posttest learning achievement was regarded as the dependent variable of the analysis. The Levene test showed that the homogeneity hypothesis was accepted, F(1, 36) = 1.69 (*p* > 0.05). In addition, F(1, 34) = 0.73 (*p* > 0.05) did not violate the assumption of regression homogeneity, and so an ANCOVA could be used to analyze the posttest scores of the two groups. Table 2 shows the ANCOVA results, F(1, 35) = 23.43 (*p* < 0.001), indicating that the DRI-based mobile professional training approach (mean = 89.72; standard deviation = 11.0) was better than the conventional technology-assisted teaching method (mean = 60.52; standard deviation = 15.0) and could effectively improve students’ learning achievement. In addition, the effect amount (η2 = 0.401) was greater than 0.138, which is a high value, indicating that the DRI-based mobile professional training approach had a significant impact on the students’ learning achievement. In other words, this learning method effectively improved the students’ learning achievement.

### 5.3. Self-Efficacy

In the study, the self-efficacy pretest questionnaire was used as a common variable, and the self-efficacy posttest questionnaire was used as the dependent variable. The Levene test was used to test the homogeneity hypothesis: F(1, 36) = 2.24 (*p* > 0.05). In addition, it was verified that F(1, 35) = 6.10 (*p* > 0.05) did not violate the assumption of regression homogeneity, and so an ANCOVA was performed to analyze the posttest results of the two groups. Table 3 illustrates the ANCOVA results, F(1, 35) = 36.66 (*p* < 0.001), indicating that the DRI-based mobile professional training approach (mean = 4.59; standard deviation = 0.52) was better than conventional technology-assisted teaching methods (mean = 3.31; standard deviation = 0.45). The adjusted average values of the experimental group and the control group were 4.48 and 3.43, respectively, which also showed that, compared with conventional technology-assisted teaching methods, the DRI-based mobile professional training approach could improve students’ self-efficacy. In addition, the effect size (η2 = 0.512) was greater than 0.138, which is a high value. This also shows that the DRI-based mobile professional training approach had a significant impact on the students’ self-efficacy. In other words, the DRI-based mobile professional training approach could effectively improve students’ self-efficacy.

## 6. Discussion

Improving students’ decision-making abilities is a crucial goal in nursing education as well as other professional training programs. It is especially important in the training program for neonatal vaccinations in Taiwan, owing to the challenge of a lack of experience dealing with practical cases. In this study, a DRI-based mobile professional training approach is proposed to cope with this problem; moreover, a quasi-experiment was conducted to assess the effectiveness of the proposed approach. The results show that this method has great potential and can improve students’ learning outcomes in terms of neonatal vaccination knowledge, self-efficacy, and learning experience. Although several previous studies have also used expert systems in educational settings, they did not guide students to make reflections by comparing their solutions with those provided by the expert systems. Instead, the previous studies mainly used expert systems as a tool for providing knowledge or feedback [41,42,43].

From a constructivist perspective, in the course of the activity, students first try to make judgments on medical cases based on their knowledge and experience and then compare them with the opinions of the expert system. This process may cause cognitive conflicts. Students should be encouraged to reflect. Many scholars have pointed out that when students are guided to reflect, they can construct their knowledge [44,45,46]. Therefore, the DRI-based mobile professional training approach for learning about neonatal vaccinations is used to assist students in answering questions raised during learning. It also guides students to think about the content of these questions from different perspectives through the process of interacting with the expert system by making decisions and comparing their decisions with those provided by the expert system. In addition, by reflecting on their answers as well as referring to the materials provided by the expert system, learners are able to connect new knowledge with the knowledge or experience they have acquired in the past [13]. This shows that the DRI-based mobile professional training approach can promote students’ self-efficacy and in-depth thinking and help them extend the scope of learning knowledge and reorganize the knowledge they have learned. Therefore, it can help students organize practical knowledge according to their learning goals. These findings are consistent with the results of Wanyan et al. [28] regarding the use of expert systems and echo the conclusions of Tang, Chang, and Hwang [19].

The results also show that the DRI-based mobile professional training approach improves students’ self-efficacy; that is, the students in this study were confident in their knowledge of neonatal vaccination and in completing the tasks assigned by the teacher. This result echoes the self-efficacy theory proposed by Bandura [47,48]. Using the DRI-based mobile professional training approach to learn about neonatal vaccination makes learning more interactive and interesting. It helps students organize and re-examine their knowledge, as proposed by Stella and Madhu [10]. This study suggests that students’ self-efficacy improves because they have the opportunity to understand the complete picture of neonatal vaccine knowledge under the expert system’s guidance and can conduct in-depth thinking by exploring relevant information on neonatal vaccination.

Accordingly, there are several practical implications from the findings of the present study. First, encouraging students to make judgments on practical cases before receiving hints or suggestions could benefit them more than directly providing solutions to them. Second, engaging students in comparing their solutions with those of the domain experts would help them make reflections and learn better. Third, using mobile expert systems in professional training has great potential for improving students’ learning performance as well as enhancing their self-efficacy.

In addition, as Taiwan has had a low birth rate in recent years and parents in Taiwan generally hesitate to have their newborns vaccinated, engaging nursing students in dealing with practicing cases in a technology-assisted interactive learning mode is very important for them to better prepare for facing practical cases. This implies that such an approach could be suitable for other countries with similar situations. On the other hand, for those countries with a high birth rate, engaging nursing students in real-life practice contexts with support from the mobile application could be an alternative.

## 7. Limitations and Future Directions

There are some limitations to this study. First, the experiment was conducted using a selected mobile application for particular nursing training content, and hence the findings might not be applicable to other applications. Second, the sample size of the study was small. Third, the experimental time was not long, and hence the results could be different if the same approach is applied to course units requiring longer learning time. Third, we only examined the effectiveness of the approach from some angles. To further investigate the effectiveness of the approach, more dimensions need to be taken into account in the future. Fourth, the learners were nursing students undergoing an internship and had not yet met the parents of newborns. This could be the reason why they did not reveal concerns about resistance from parents of newborns in the interviews.

On the other hand, from the experimental results, a statistically significant difference was found between the two groups despite the small sample size; moreover, the effect size was very good. This implies that the use of the expert system as an assistant to guide learners to practice making decisions and reflections and to consider the answers to a question from diverse perspectives has great potential in professional training. Therefore, it is worth conducting follow-up experiments in the future.

Based on the results and discussion of the research, the following suggestions are proposed for future research:

(1) When studying DRI-based professional training, personal factors such as individual students’ preferences or self-efficacy might also affect the training outcomes. Taking these factors into account could further improve trainees’ performance. Therefore, it is suggested that researchers take personal factors into account when designing their future studies.
(2) The effectiveness of DRI-based professional training can be further explored from different angles. For example, in the application of neonatal vaccination, up-to-date information (e.g., regarding the COVID-19 pandemic) can be added to the learning content of the training program to enable the trainees to link what they have learned to practical clinical situations.
(3) The use of an expert system as part of the learning method has good potential for inspiring students to think deeply, increasing their critical thinking, enhancing their reflective ability, promoting their professional capacity to deal with problems related to neonatal vaccination for mothers and families in their future workplaces, and promoting their willingness to further actively explore the relevant knowledge. Therefore, it is suggested that researchers investigate the diverse effects of the approach on trainees’ performance.
(4) The DRI-based professional training can be applied to other medical professional training courses or even other professional training programs to explore trainees’ decision-making and problem-solving performance in different application contexts.

## 8. Conclusions

The main contribution of the current research is to propose an innovative education model and verify its effectiveness through experimental data. Tang et al. [19] pointed out that the experimental design of the application of expert system learning combined with natural language processing and analysis technology is relatively insufficient in medical procedures compared with other fields. Therefore, it is essential to determine how to use expert systems for interdisciplinary cooperation and to develop more medically-assisted expert learning systems through the cooperation of computer, mathematics, engineering, education, and medical professionals.

As the mobile devices used in this study were the participants’ own smartphones and the data adopted in the mobile system was from the Health and Welfare Department of Taiwan, the cost of applying the mobile application in professional training was relatively low in comparisons with developing a new system with a new database. Moreover, using open sources not only reduces the implementation cost but also promotes the usage of the sources. Therefore, it is suggested that, in the future, similar approaches can be considered for other professional training programs.

## Figures and Tables

**Table 1:** Themes, coding items, and number of occurrences of the interview results.

Theme	Code	The Number of Times Mentioned	
		Experimental	Control
Increase the value of activities	Find their own steps through their own practice process.	6	3
	Identify their own steps through the judgment tasks.	6	3
	Have the teacher help find their steps by uploading their practice.	6	5
Enhance the planning and expensive capacity of conspicuous approaches	Enhance the capability to plan conspicuous approaches through practice.	5	0
	Increase the expanse of practice for conspicuous approaches by demonstrating their practice.	5	0
	Enhance the scheduling and valuable capability of conspicuous approaches through the learning platform.	6	0
Promote decision making [38]	Decision on their conspicuous approaches through the learning platform.	6	4
	Decision on their conspicuous knowledge through the learning platform.	6	4
Improve the learning reflection [39]	Increase the efficiency of reflection during in-class practice.	6	5
	Consider the scenarios that could occur in class.	6	4
	Increase the understanding of neonatal vaccination knowledge.	6	4
	Increase the time for practicing.	6	3
Providing students with personalized interaction [40]	Allowed to repeat practice.	6	4
	Acceptable to review.	6	3
	Acceptable to learn anytime.	6	2

**Table 2:** ANCOVA study results for the experimental group and the control group.

Variable	N	Mean	SD	Adjusted Mean	Std. Error.	F	η2
Experimental group	20	89.72	11.00	87.90	3.25	23.43 ***	0.401
Control group	18	60.52	15.00	62.53	3.47		

*** *p* < 0.001.

**Table 3:** ANCOVA self-efficacy analysis results for the experimental group and control group.

Variable	N	Mean	SD	Adjusted Mean	Std. Error.	F	η2
Experimental group	20	4.59	0.52	4.48	0.11	36.66 ***	0.512
Control group	18	3.31	0.45	3.43	0.12		

*** *p* < 0.001.

## Data Availability

No datasets were used in the present research.
